# Auditory and tactile frequency mapping for visual distance perception: A step forward in sensory substitution and augmentation

**DOI:** 10.1371/journal.pone.0318354

**Published:** 2025-03-03

**Authors:** Pingping Jiang, Jonathan Rossiter, Christopher Kent

**Affiliations:** 1 School of Engineering Mathematics and Technology, University of Bristol, Bristol, United Kingdom; 2 SoftLab, Bristol Robotics Laboratory, Bristol, United Kingdom; 3 School of Psychological Science, University of Bristol, Bristol, United Kingdom; Cook Childrenś Health Care System: Cook Children’sMedical Center, UNITED STATES OF AMERICA

## Abstract

Vision is crucial for daily tasks and interacting with the environment, but visual impairment can hinder these activities. Many sensory substitution products and studies prioritize providing abundant and accurate information, yet often overlook the inherent relationship between different modalities, potentially preventing users from receiving information intuitively. This study investigated the representation of visual distance using auditory and vibrotactile frequency through a series of psychological cross-modal matching experiments. By establishing mapping functions between auditory/vibrotactile frequency and visual distance, we aim to facilitate the design of sensory substitution devices that take visual distance information (ranging from 1 m to 12 m) and convert it into non-visual information (auditory frequency within the range 47-2764 Hz or vibrotactile frequency within the range 10–99 Hz). Results show distinct patterns regarding the correlation between visual distance and frequency in both auditory (auditory frequency-to-visual distance) and vibrotactile (vibrotactile frequency-to-visual distance) domains. The prevailing trend (59%) was a monotonic negative correlation (i.e. higher frequencies are associated with shorter distances), while 24% of participants demonstrated a consistently positive correlation. Additionally, we compare this study with our previous investigations into the reverse cross-modal mapping of visual distance-to-auditory frequency and visual distance-to-vibrotactile frequency. We reveal common patterns between these two studies (negative and positive correlations), suggesting a bidirectional mapping between visual distance and frequency in both auditory and vibrotactile domains, and the potential for new sensory substitution devices for those with visual impairment by integrating underlying cross-modal mechanisms to enhance intuitive and natural human-machine interaction.

## Introduction

In many everyday situations, vision, sound, and touch are seamlessly integrated to form a coherent perception of the world. However, when one sense is lost (e.g. through degradation, interference, or physical loss) other senses must be relied upon to either substitute or compensate for the lost sense. There are more than 314 million individuals who experience various degrees of pervasive visual impairment and among them, around 45 million are classified as blind [1]. Individuals with severe visual impairments usually rely more on auditory or haptic cues to gather information about their environment. There are several widely used tools for people with visual impairments, ranging from Braille displays which convert digital text into tactile information [2,3], allowing users to read through touch, to screen readers which audibly convey digital information to users [4,5]. Tools such as white canes help detect obstacles or guides on the ground, while navigation apps with auditory instructions guide users through unfamiliar surroundings [6–8]. These devices are primarily focused on addressing the specific challenges faced by blind individuals, such as accessing information, navigating environments, and performing daily tasks independently.

### Sensory substitution technology

Currently, there is a diverse range of sensory substitution devices designed specifically for the visually impaired. These devices serve the dual purpose of helping the user to navigate their surroundings and to access information effectively. For instance, the Maptic [9] device incorporates a camera, Google Maps, and GPS receiver to achieve turn-by-turn navigation through vibration at the left or right side of the body; Microsoft Soundscape [10] explored the use of audio-based technology to enable people to build a richer awareness of their surroundings using headphones or earbuds; BrainPort [11] takes a unique approach by translating digital information from a wearable video camera into gentle electrical stimulation patterns on the surface of the tongue; Envision Glasses [12] extract information from images and describe the images out loud so the user has a greater understanding of the environment around them. In addition to these practical devices, diverse research is dedicated to developing navigation solutions for individuals with visual impairments. These efforts encompass an array of sensors [13–15] (including cameras, accelerometers, and gyroscopes [16], etc.) as well as various feedback approaches such as auditory feedback [17,18], haptic feedback [19–22], or a combination of both [23].

The essence of these sensory substitution devices revolves around two key elements: 1. the integration of sensors, and 2. providing informative feedback in a different sensory modality. A greater number of sensors typically results in a more comprehensive data set, but at the expense of increased signal processing and time and energy costs. An optimal approach would involve fewer sensors that offer concise yet accurate sensing capabilities. The conventional means of delivering feedback rely on either auditory cues or tactile sensations, sometimes combining both. Auditory feedback typically includes pre-saved guidance for a specific contextual environment while tactile feedback – often in the form of vibrations – is used to convey directional information or warn of potential obstacles. However, the underlying mechanisms of this sensory substitution and augmentation are not fully known. Therefore further investigation is warranted into how sensory information from one modality is conveyed in another from a psychological perspective, with the aim of delivering intuitive, fast, and eﬃcient information gathering and communication, such as when danger is present. The key feature (e.g. danger) needs to be expressed and understood quickly without lengthy and complex learning and training processes. As reported in [24], human-machine interfaces that consider cross-sensory mappings and user perceptual knowledge can enhance eﬃciency in interpreting and interacting with information. Implementing stimuli connected to intrinsic cross-modal mapping can contribute to achieving effective sensory substitution [25]. Therefore, cross-modal research on sensory substitution devices (SSDs) holds the potential for improving natural and intuitive human-machine interactions.

### Cross-modal matching in sensory substitution of distance

Research into cross-modal matching has a long-established history in psychology [26], involving the study of consistent mappings between attributes or dimensions of stimuli across different sensory modalities. This area includes various studies such as sound and vision matching [27], sound and taste matching [28], vision and taste matching [29], haptic and sound matching [30], haptic and vision matching [31,32], among others [33,34]. The auditory pitch has been widely studied in cross-modality research including its correspondence with visual features including vertical location, size, spatial frequency and contrast [35]. These congruent associations have been shown to enhance perceptual performance in both sighted and visually impaired individuals [36]. As highlighted in our prior investigation [37], vision-to-sound and vision-to-haptic cross-modal matching studies collectively indicate that the correlation between vision and the two senses of hearing and touch is widespread. In the realm of perceptual cognition, the sensory systems serve as vital pathways through which individuals acquire essential information concerning the spatial localization of events within the surrounding environment. Distance perception is also an important – but largely missing – element in crafting immersive virtual experiences that replicate the real world. The translation of visual distance carries considerable importance, as it may enable people to naturally and quickly grasp and represent distances they cannot see through auditory cues or vibrations.

While both sight and hearing play crucial roles in estimating the distance to an object (e.g., through visual size and sound volume [38]), cross-modal research in the perception of distance remains limited. In the work by Maidenbaum et al. [39] and Chebat et al. [40], distance information was translated into vibrotactile and auditory frequency cues to provide real-time feedback to participants navigating through virtual mazes. However, in these studies, there was a lack of consideration and measurement of specific cross-modal projection relationships. Another significant aspect that has been overlooked is the assessment of individual differences in the perception of alternate sensory modalities as our previous study highlighted [37]. For instance, in [41], they investigated the effectiveness of five depth sonifications (frequency, amplitude, reverberation, the repetition rate of sounds, and signal-to-noise ratio) in assisting the design of SSDs for blind people within the range of 0-1 m by assuming a linear relationship between the sonification parameter and the distance without providing a theoretical grounding. Individual differences in perception can significantly impact the effectiveness of sensory substitution technologies; what works well for one person may not work as effectively for another due to variations in sensory acuity, cognitive processing, or emotional state. Thus, a device that takes advantage of, or aligns with, the natural sensory connections inherent in the human sensory system is likely to be most effective.

Our approach to cross-modal matching is noteworthy for its fundamental one-dimensional nature. While many prior SSDs offer various streams of concurrent information, they often necessitate participants to learn or undergo training to comprehend the connections. In our study, we prioritize establishing an intuitive mapping between modalities, emphasizing a natural and swift connection. This focus on intuitiveness is particularly relevant in time-critical situations [42]. It is important to recognize the inherent trade-off between the complexity/fidelity of the representation and the speed/ease of interpreting the mapping. In our specific context, we aimed to comprehend the fundamental one-dimensional functions mapping visual distance to auditory and vibrotactile frequency.

### Aim of study

**Fig 1:**
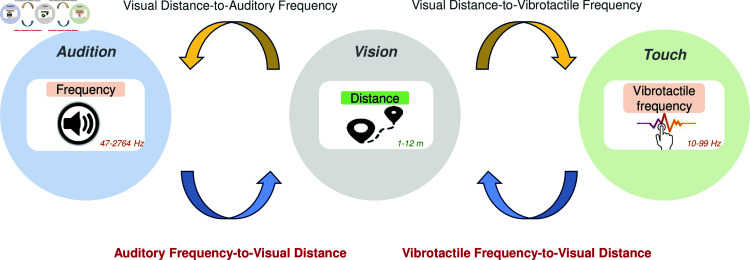
Experiment demonstration of this study (forward, blue mappings) and previous study [37] (inverse, yellow mappings).

In previous study [37] in which 101 participants mapped the distance (represented by an image of a model at that distance) to an auditory or vibrotactile frequency via adjusting a virtual tuning knob, we investigated how frequency can serve as a means to map distance (in the range of 1–12 m) from the visual domain into the tactile and auditory domains where the frequency range was 10–99 Hz for tactile frequency and 47–2764 Hz for audio pitch (as shown in Fig 1). Our findings revealed robust cross-modal correlations for both the mapping of visual distance to auditory frequency and the mapping of visual distance to vibrotactile frequency. Over 70% of the participants exhibited a consistent negative relationship (longer distance corresponding to lower auditory or tactile frequency), while no more than 10% exhibited a consistent positive relationship (longer distance corresponding to higher auditory or tactile frequency) between the variables. These results revealed a strong cross-modal sensory correlation that holds potential for advancing sensory substitution technologies and developing devices to augment human perception. The results also highlighted the presence of individual variations in comprehending distance information from alternative modalities.

Here, in contrast, we study the forward auditory frequency-to-visual distance and vibrotactile frequency-to-visual distance cross-modal matching (see Fig 1) and compare these outcomes with our previous study’s findings (visual distance-to-auditory frequency and visual distance-to-vibrotactile frequency). This comparison aims to explore the full bidirectional sensory mapping relationships, enhancing our understanding of the cross-modal mapping process and its potential applications in multisensory contexts. In this research, we employed a methodology similar to [37] where four experiments were conducted to examine various cross-modal matching scenarios. Instead of viewing a visual distance and then turning a dial to select the frequency, participants were played a random frequency and turned a dial to adjust the distance. In the auditory frequency-to-visual distance matching task, participants heard a tone of a single frequency, while in the vibrotactile frequency-to-visual distance matching task, participants felt a single vibration frequency on their fingers. The participant then adjusted a dial to select a matching distance as represented by images of objects placed at various distances (1–12 m). Based on [37], we expect to find both negative and positive relationships.

## Methods

The primary objective of this study was to explore the mapping relationships from auditory and vibrotactile frequencies to visual distance. Following [37], we conducted four experiments as shown in Table 1. The table details the experimental design, where Model Type distinguishes between two categories presented in the images: humans and inanimate objects, with three instances of each type. The experiments were conducted between July 13th and September 16th, 2022. All participants provided informed consent through our online experiment platform, which included a digital information sheet and consent form. This study was approved by the Faculty of Engineering Research Ethics Committee at the University of Bristol (study reference number: 9402).

**Table 1:** Summary of experiments conducted in the study.

Experiment	Mapping Relationship	Model Type	No. of Participants	Age Range	Mode
**1a**	Auditory Frequency to Visual Distance	Human	25	18-49	Online
**1b**	Auditory Frequency to Visual Distance	Inanimate Object	25	18-49	Online
**2a**	Vibrotactile Frequency to Visual Distance	Human	21	18-45	In Person
**2b**	Vibrotactile Frequency to Visual Distance	Inanimate Object	22	18-45	In Person

### Participants

Participants were recruited through two different approaches: the audio experiments (1a and 1b) were conducted online and participants were recruited from Prolific [43], an online recruitment platform; the vibrotactile experiments (2a and 2b) were run in person, and participants (with age between 18 and 45) were recruited from the University of Bristol in return for gift vouchers. In Experiments 1a and 1b, participants ranged in age from 18 to 49, were fluent English users, had self-reported normal or corrected-to-normal vision, and had no known hearing impairments. In Experiments 2a and 2b, apart from the previously mentioned criteria, participants were also required to ensure the availability of both hands for the experiments.

### Materials

**Fig 2:**
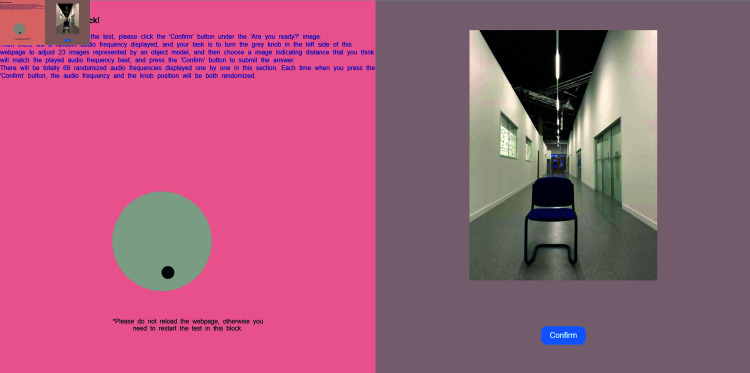
Experiment webpage for frequency-to-visual distance matching. A user-adjustable dial on the left is rotated by the participant to switch the image on the right until its distance matches the auditory or vibrotactile frequency being given to the participant.

**Fig 3:**
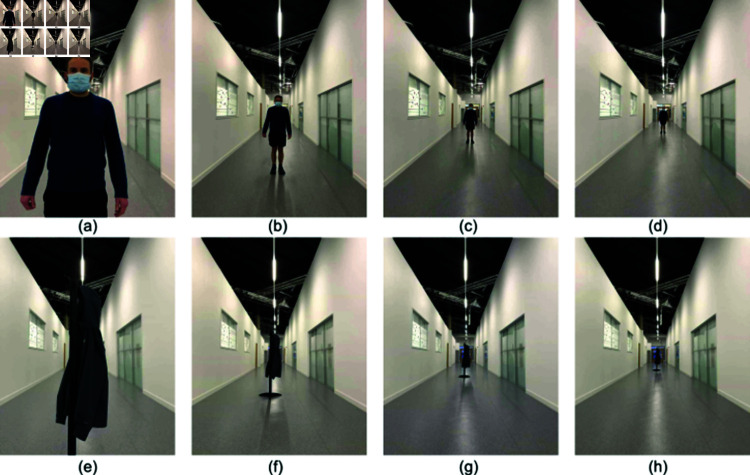
Example image stimuli at different positions. (a) Human at 1 m position;(b) Human at 4 m position; (c) Human at 8 m position; (d) Human at 12 m position; (e) Coat stand at 1 m position; (f) Coat stand at 4 m position; (g) Coat stand at 8 m position; (h) Coat stand at 12 m position.

A set of custom-written webpages (one of them illustrated in Fig 2) were employed to administer all the experiments. The independent variable in all experiments was frequency (auditory frequency for the audio-to-visual distance experiments 1a and 1b, and vibrotactile frequency for the vibrotactile to visual distance experiments 2a and 2b) and the dependent variable was the distance the item appeared away from the current viewpoint. In Experiment 1a and 2a, distance was represented by images of three human models, and in Experiment 1b and 2b distance was represented by images of three inanimate objects (a coat rack stand as shown in Fig 3, a chair, and a signpost). Visual distance images with a size of 600  ×  450 pixels were the same set as defined in [37] and consisted of photos of human or object models in a well-lit corridor, at a measure distance from the camera in the range of 1–12 m in increments of 0.5 meters.

Participants undertook the online tasks of Experiments 1a and 1b – auditory frequency-to-visual distance matching – using their personal computers. The auditory tones were sine waves transmitted through the participants’ individual audio output devices. Participants could use speakers or headphones. The participants set the loudness to a comfortable audible level at the start of the experiment and were instructed to leave it at this level for all tasks. It is worth noting that there may be limitations associated with the hearing requirements and device control in the online auditory experiments where participants were recruited worldwide from online platforms.

For Experiment 2a and 2b – vibrotactile frequency-to-visual distance matching – participants conducted the experiment in a quiet laboratory setting, seated in front of a 21-inch LCD monitor and maintained an approximate viewing distance of 50 cm from the monitor. Vibrotactile stimuli were played through the Tactor (a vibrotactile electromagnetic solenoid-type stimulator) made by Dancer Design. The Tactor was attached to the index finger of the participant’s non-dominant hand. 69 vibrotactile frequencies were uniformly selected across the [10, 99] Hz range, with the tactile signal being a sine wave. These frequencies were used as vibrotactile stimuli in both Experiment 2a and 2b. This frequency interval was determined to maintain a relatively consistent stimulation force on the fingertip, rather than focusing on the amplitude of the movement. The amplitude is influenced by the force as well as other factors like the mechanical load and the constraints on the solenoid’s movement. In our study, due to individual differences among participants, the amplitude cannot be directly measured. During the trials, participants were instructed to place the hand with the Tactor facing downwards on a table. The hand was wrapped with a towel to minimize any noise or distractions caused by the vibrations. Additionally, participants were provided with noise-cancelling over-ear headphones to wear throughout the experiment.

### Procedure

In Experiment 1a and 1b, participants were played 69 auditory frequencies in random order, evenly distributed within the range [47, 2764] Hz, and were required to turn the virtual dial on the web page to choose the image corresponding to the distance that matched the auditory frequency. Experiments 2a and 2b were identical to Experiment 1a and 1b, frequencies were played in a randomized order through the finger-mounted Tactor, allowing participants to feel the vibrotactile sensation generated.

Following an information screen, participants were introduced to a preview of the visual stimuli. This preview showed a series of 23 images selected by manipulating the on-screen dial. To interact with this dial, participants used a mouse as their input device. For an increase in visual distance, participants held down the left mouse button while turning the dial clockwise, resulting in a change in image to an object/human further away. Conversely, turning the dial counter-clockwise led to a decrease in visual distance, resulting in a change in image to an object/human closer to the camera. Throughout the study, participants could manipulate the dial freely. Once the maximum or minimum visual distance was reached, the dial became fixed in that direction. Participants could then rotate the dial in the opposite direction, restarting the selection process in reverse, until they were content with their response for the current frequency. This approach was akin to the functioning of a dial such as a rotary volume control, providing participants with a tangible comprehension of the distance range involved in the task.

Participants were then guided to a preview webpage where they could preview the 12 auditory pitches or tactile vibrations, starting from lower frequencies and progressing to higher frequencies. The auditory or vibrotactile stimuli were automatically played upon entering this preview webpage, and participants could replay them by clicking the ‘play again’ button. After participants became familiar with the task, they completed the online consent form and commenced the experimental trials.

For each participant, the experiment consisted of three identical blocks, and each block consisted of 69 trials. These trials included the presentation of 69 distinct auditory or vibrotactile frequencies in a randomized sequence, along with images of either 3 human models (Experiment 1a and 2a) or 3 object models (Experiment 1b and 2b) at 23 different distance positions. During each trial, the images representing distance appeared on the right side of the webpage, while the response dial was positioned on the left. At the start of every trial, the position of the dial was reset randomly. This ensured that participants did not anchor their responses to any specific portion of the visual distance spectrum. At the outset of each trial, no images relating to distance were displayed. Instead, a prompt image served as the default display, encouraging participants to rotate the dial. After choosing the intended visual distance response, participants proceeded by clicking the ‘Confirm’ button’ (as shown in Fig 2), triggering the next randomized auditory or vibrotactile frequency along with the display of the prompt image. At the end of a block, participants were allowed a short break before completing the next block. Overall, the experiment (207 trials) lasted approximately 30 mins.

## Results

**Fig 4:**
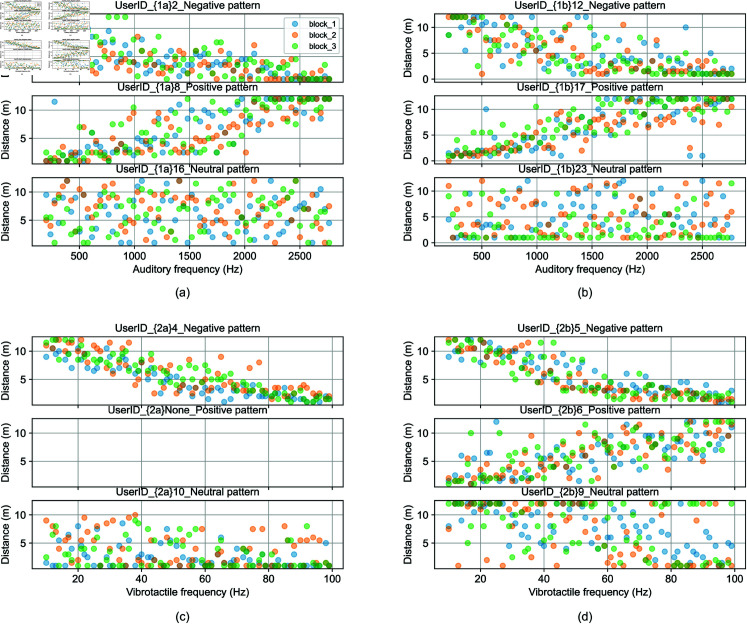
Perceived distance as a function of auditory or vibrotactile frequencies, example raw data responses from different participants. (a) Experiment 1a, three patterns: negative, positive, and neutral. (b) Experiment 1b, three patterns: negative, positive, and neutral. (c) Experiment 2a, two patterns: negative, and neutral. (d) Experiment 2b, three patterns: negative, positive and neutral.

The analysis of experimental data adopted a method similar to our previous study [37]. First, visual distance against auditory and vibrotactile frequencies was plotted (examples shown in Fig 4). It was found that while most participants showed a negative relationship (closer distances were associated with higher frequencies), not all participants followed this pattern. Some showed a positive relationship (closer distances were associated with lower frequencies) while others appeared to exhibit more random response patterns. In the subsequent analysis, we will examine the variability within each category (comparing the three human models or the three inanimate models) and analyse the differences between the two categories (human versus inanimate models).

*Within-group effects analysis to examine whether participants’ responses were influenced by three factors: experimental blocks, instances of human model type, and instances of inanimate model type.* For each participant, we computed linear slope values (distance by frequency) across blocks and model instances, then conducted repeated measures ANOVAs. Across all experiments, we found no significant effects: block comparisons yielded p-values ranging from 0.088 to 0.847, and model instance comparisons yielded p-values ranging from 0.278 to 0.788 (Experiment 1a: F(2,48)=0.295,p=0.746; Experiment 1b: F(2,48)=1.313,p=0.278; Experiment 2a: F(2,40)=0.239,p=0.788; Experiment 2b: F(2,42)=1.141,p=0.329). Since neither blocks nor model instances significantly influenced the data, we proceeded with the primary analysis by averaging across both factors.

**Fig 5:**
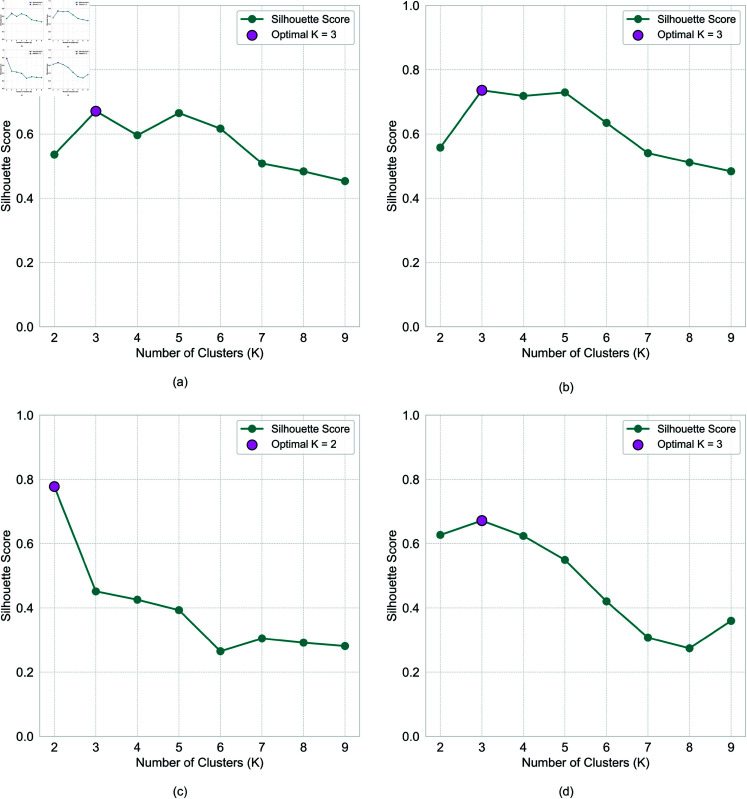
Optimal number of clusters determined using the silhouette score. For Experiment (a) 1a, (b) 1b, (c) 2a, (d) 2b - the silhouette score method was applied to identify the optimal K values for K-means clustering.

**Fig 6:**
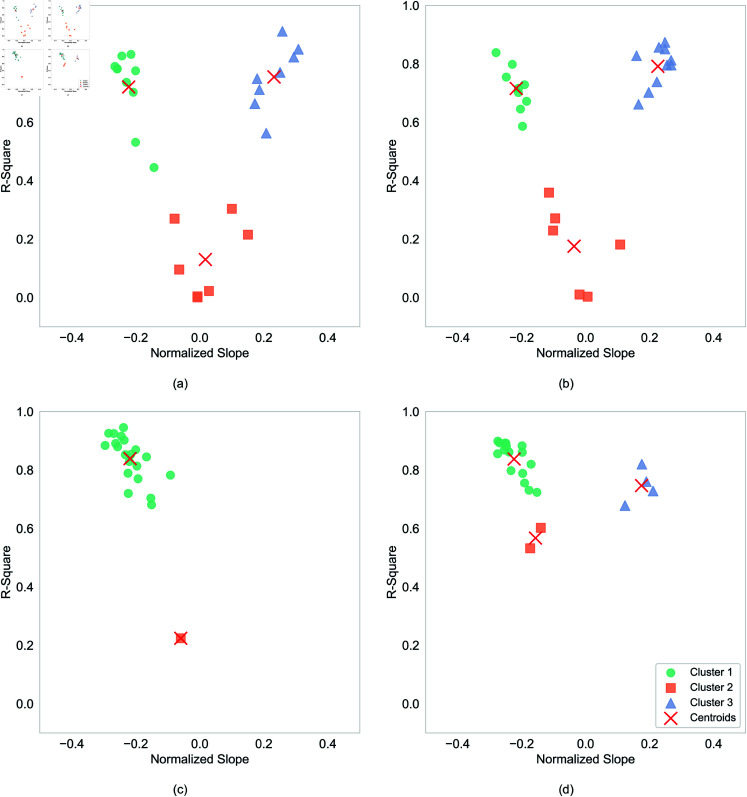
Data partitioning into clusters. For Experiment (a) 1a, (b) 1b, (c) 2a, (d) 2b respectively, K-means clustering was applied based on the optimal number of clusters (K) from Fig 5. Each marker represents a single participant.

*Data clustering to identify typical patterns.* To more objectively classify the direction of the relationship between distance and frequency for each participant, a linear model was first fitted to their individual data. The data were then categorized into three groups based on two criteria: the slope value (*b*), which can be a positive or negative, and $R^{2}$ value. K-means clustering was applied to the $R^{2}$ and normalized slope data for each experiment to identify distinct clusters. The Silhouette Score method was utilized to determine the optimal number of clusters, as illustrated in Fig 5. The resulting cluster centers are shown, with data points in Fig 6. We see that the data is naturally grouped into three (or fewer) clusters, which correspond to negative correlation (upper left quadrant) , positive correlation (upper right quadrant), and random distribution (lower half). This categorization is consistent with the scatter plots presented in Fig 4). Our analysis focuses on the data exhibiting non-zero slopes and higher $R^{2}$ values, specifically negative and positive correlations. For instance, in Experiment 1a (Fig 6(a)), Cluster 3 displays a slope greater than zero, indicating a positive relationship between distance and frequency, while Cluster 1 exhibits a negative correlation. Conversely, in Cluster 2, the slope is near zero, suggesting no clear correlation between distance and frequency, and the lower $R^{2}$ values indicate random noise. Consequently, this data was excluded from further analysis. Fig 6 illustrates the cluster analysis results across all four experiments„ and Table 2 summarizes the proportions of negative, positive, and neutral correlations among users for each experiment.

**Table 2:** Proportions of correlations across users.

Experiment	Negative Correlation	Positive Correlation	Neutral Correlation
**1a**	40.0%	32.0%	28.0%
**1b**	36.0%	40.0%	24.0%
**2a**	95.2%	–	4.8%
**2b**	72.7%	18.2%	9.1%

*Fitting curves to mathematical models:* We quantified, in an exploratory analysis, the nature of the function relating distance to frequency for each user in two groups that exhibited a consistent relationship (either positive or negative). A variety of functions were fit to each individual’s dataset and averaged across model and block. We identified that the commonly used mathematical functions in psychophysics that capture a wider range of possible shapes [44,45], such as linear (), exponential (), power (), and natural logarithmic (), can be used to approximate the data. The goodness-of-fit of these fitting functions was assessed by comparing the $R^{2}$ values, RMSE (Root Mean Square Error) values and accompanying variability, enabling us to evaluate and determine fitting function for each user and correlation.


$$\begin{array}{c} y_{linear}=b\cdot x+a \end{array}$$ (1)

$$\begin{array}{c} y_{exponential}=a\cdot e^{bx} \end{array}$$ (2)

$$\begin{array}{c} y_{power}=a\cdot x^{b} \end{array}$$ (3)

$$\begin{array}{c} y_{natural\text{\_}log}=a+b\cdot \ln(x) \end{array}$$ (4)

*Individual curve fitting performance: Comparison of $R^{2}$ and RMSE values across four mathematical models.* In Experiment 1 (including both 1a and 1b), negative correlations were observed in the data of 19 participants, while 18 participants exhibited positive correlations. In Experiment 2 (including both 2a and 2b), 36 participants demonstrated negative correlations, and 4 participants showed positive correlations. For each participant, we compared the $R^{2}$ and RMSE values to identify the optimal fit (a higher $R^{2}$ value and lower RMSE value indicate a better fit between the model and the data). The distribution of the best-fitting models is shown in Table 3. In the negative correlation group of Experiment 1, the natural logarithmic and exponential functions each provided the best fit for 42.1% of participants. For the positive correlation group in Experiment 1, the power function offered the best fit for 38.9% of participants, while the natural logarithmic function was optimal for 33.3% of participants. In Experiment 2 negative correlation group, most participants (52.8%) had data best represented by a linear function, while the exponential model was the second most preferred, fitting 38.9% of participants. In the positive correlation group of Experiment 2, the four mathematical models fit equally well, with each model being optimal for 25% of participants.

**Table 3:** Proportions of best-fitting models across users.

Model	Negative Correlation (Exp 1)	Positive Correlation (Exp 1)	Negative Correlation (Exp 2)	Positive Correlation (Exp 2)
**Linear**	10.5%	22.2%	52.8%	25.0%
**Natural Logarithmic**	42.1%	33.3%	8.3%	25.0%
**Power**	5.3%	38.9%	–	25.0%
**Exponential**	42.1%	5.6%	38.9%	25.0%

**Fig 7:**
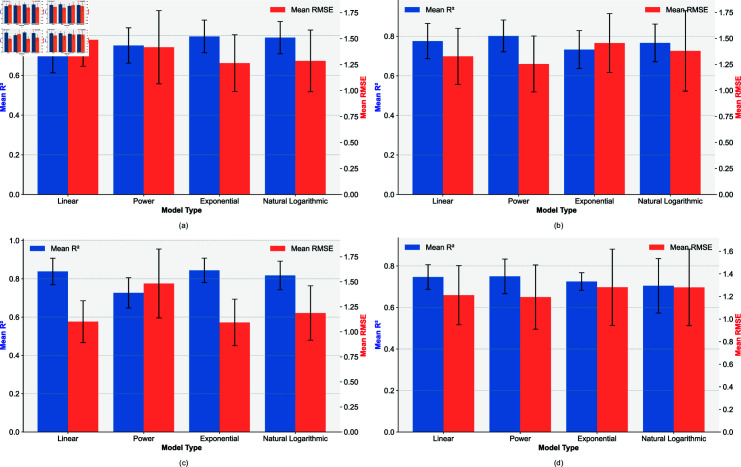
Average $R^{2}$, RMSE, and standard deviation of model fit across users. (a) Negative correlation group in Experiment 1, (b) Positive correlation group in Experiment 1, (c) Negative correlation group in Experiment 2, (d) Positive correlation group in Experiment 2.

*Group curve fitting performance: Mean $R^{2}$, Mean RMSE values, and associated variability across users for each correlation group.* Fig 7 illustrates that, in Experiment 1 (Fig 7(a)), the exponential model provided the best fit for the negative correlation group, with a mean $R^{2}$ of 0.7963 (SD = 0.0822) and a mean RMSE of 1.2646 (SD = 0.2734). In contrast, for the positive correlation group in the same experiment (Fig 7(b)), the power model outperformed others, yielding a mean $R^{2}$ of 0.8016 (SD = 0.0802) and a mean RMSE of 1.2547 (SD = 0.2687). Similarly, in Experiment 2 (Fig 7(c)), the exponential model again was the best fit for the negative correlation group, with a mean $R^{2}$ of 0.8440 (SD = 0.0639) and a mean RMSE of 1.0983 (SD = 0.2327). For the positive correlation group in Experiment 2 (Fig 7(d)), the power model showed the best performance, achieving a mean $R^{2}$ of 0.7498 (SD = 0.0834) and a mean RMSE of 1.1945 (SD = 0.2844).

In this study, we propose a representative fitting function for each correlation group, informed by both individual- and group-level curve fitting performance. Although these functions may not be optimal, they provide a practical and eﬃcient approach with potential real-world applications. For the negative correlation group in Experiment 1, the natural logarithmic and exponential models were equally matched to participants based on individual best-fit results. However, when considering group-level performance, the exponential model showed superior average fitting results. In the positive correlation group of Experiment 1, the power model emerged as the best, both in terms of the highest proportion of individual best fits and in overall group curve fitting performance. In Experiment 2 negative correlation group, the linear model most frequently matched to individuals, but the exponential model demonstrated the best group-level fit. However, the group-level performances of the linear and power models were very similar, with differences in average $R^{2}$ and RMSE of less than 0.006 and 0.009, respectively. Therefore, the linear model was selected to represent this group. In the positive correlation group of Experiment 2, since all four models matched equally to participants, the group-level performance was used to determine the representative model, with the power model showing the best fit.

**Fig 8:**
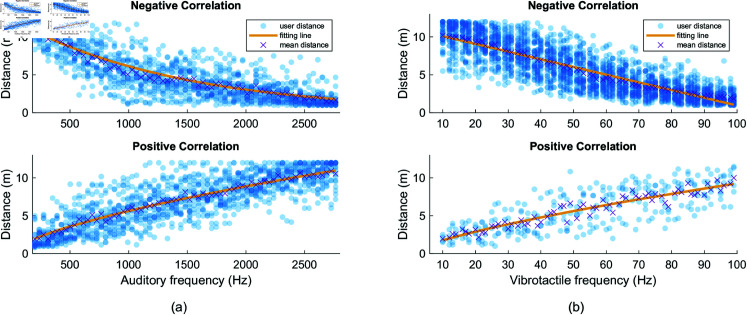
Plot of representative function for each correlation group. (a) Negative and positive correlation between auditory frequency and visual distance in Experiment 1. (b) Negative and positive correlation between vibrotactile frequency and visual distance in Experiment 2.


*Plot of representative function for each correlation group:* In this section, the parameters *a* and *b* for the fitting function were derived from the combined frequency and distance data of all users in each correlation group. This involved pooling the data from all users in a given correlation group and performing curve fitting on the aggregated dataset. The exponential model, representing the negative correlation in Experiment 1, is shown in and Fig 8(a), while the power model, capturing the positive correlation in Experiment 1, is depicted in and Fig 8(a). For Experiment 2, the linear model representing the negative correlation is illustrated in and Fig 8(b), and the power model, which describes the positive correlation, is shown in and Fig 8(b).

$$\begin{array}{c} y_{exp,negative}=12.3319\cdot e^{-0.0007x} \end{array}$$ (5)

$$\begin{array}{c} y_{power,positive}=0.0606\cdot x^{0.6561} \end{array}$$ (6)

$$\begin{array}{c} y_{linear,negative}=11.1098-0.1016x \end{array}$$ (7)

$$\begin{array}{c} y_{power,positive}=0.3318\cdot x^{0.7232} \end{array}$$ (8)

The model-based analysis presents a complex picture, with no single function emerging as the best fit for all the data. Further studies will be required to replicate and expand upon these findings before definitive conclusions can be made. However, this exploratory analysis highlights the need for detailed behavioural investigations to understand the underlying functions more clearly.

## Discussion

**Fig 9:**
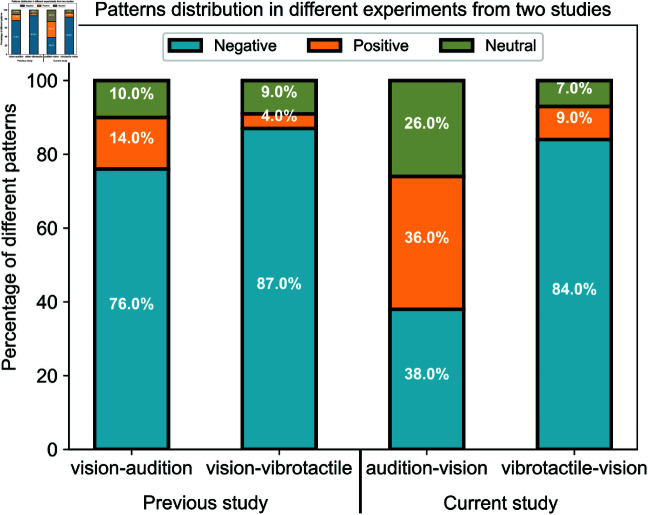
Patterns illustrating the correlation between visual distance and frequency across audio and vibrotactile domains obtained from all experiments conducted in both the present (forward) and prior (inverse) studies [37].

This study has found that there were two predominant patterns of mapping in both auditory frequency-to-visual distance and vibrotactile frequency-to-visual distance experiments. These functions remain notably consistent across various visual image models and among participants, where participants’ judgments regarding mapping distance and frequency are unaffected by the image models employed. In this study, as shown in Fig 9, for auditory frequency-to-visual distance experiments (Experiments 1a and 1b) it was observed that 38% consistently exhibited a negative correlation between auditory frequency and visual distance perception, while 36% consistently showed a positive relationship. In the vibrotactile frequency-to-visual distance experiments (Experiments 2a and 2b), the results were particularly striking. A clear majority, 84% of participants, demonstrated a consistently negative correlation between vibrotactile frequency and visual distance. This suggests that for the majority of participants, the increased vibrotactile frequency was associated with a shorter visual distance, in contrast, only 4 participants (9% of vibrotactile-to-vision experiments) displayed a consistently positive relationship, indicating that higher vibrotactile frequencies were linked to further perceived visual distances for this smaller subset. It is important to highlight that in the auditory frequency-to-visual distance experiments, the neutral pattern (where no pattern is found) accounts for a significant 26%, which is notably larger when compared to the neutral pattern proportion in the vibrotactile frequency-to-visual distance experiments (7%), the previous visual distance-to-auditory frequency experiments (10%) [37], or the previous visual distance-to-vibrotactile frequency experiments (9%) [37]. The significant difference in these percentages may be attributed to the diversity of the participant pool. Specifically, participants for the auditory frequency-to-visual distance experiments were recruited from a global online platform, resulting in a diverse sample, whereas participants for the other three types of experiments were sourced exclusively from the University of Bristol. This may suggest that individual differences could affect the perception process. Meanwhile, the significant portion of neutral relationships observed in these experiments may stem from the unsupervised nature of the experimental process, wherein participants may not have been attentive or committed to the task.

It is useful to compare the results from this study with our previous findings [37]. As observed, the major pattern that arose from data analysis is the negative correlation, accounting for around 70% of all results from both studies. Nevertheless, the significance of the positive correlation pattern, which accounts for approximately 16%, cannot be overlooked, particularly when considering the substantial 36% observed in the auditory frequency-to-visual distance experiment (see Fig 9). There are many similarities between the two studies; for instance, experimental outcomes remained unaffected by variations in image models, and consistent results were observed across several rounds of repetition tests. Both studies yielded two notable relationships: positive and negative, with the negative relationship being dominant. However, there are also differences: in the current study, curves exhibit a steeper slope compared to the previous study across both auditory and vibrotactile domains.

The cross-modal mapping presented here offers valuable insights into the connection between frequency and distance across vision, auditory perception, and tactile sensations. Our experiments reveal a negative relationship in both auditory and vibrotactile contexts. This pattern in auditory experiments may be explained by findings from Parise et al. [46], which show statistical regularities in natural auditory environments and examines how these regularities shape the perceptual mechanisms underlying spatial hearing, suggesting that the filtering properties of the outer ear may have evolved to mirror the statistics of these environments. While their research focuses on elevation, it is reasonable to speculate that similar filtering properties apply to hallways in terms of depth. Hallways can be considered low-pass filters, where distant sound sources have less high-frequency content. Given frequent exposure to hallways, people may have learned to associate higher frequencies with nearer distances and lower frequencies with further distances. This learned relationship makes it unsurprising that individuals intuitively link higher frequencies with proximity and lower frequencies with greater distances. Extending our investigation to include this aspect could provide valuable insights into how spatial acoustics influence perceptual mappings in different environments. Meanwhile, the intriguing presence of both negative and positive correlations indicates the underlying monotonic cross-modal relationship holds, but its polarity is subject to individual variations. This finding highlights personalized cross-modal matching and this work aims to advance the field of assistive technology, ensuring that SSDs not only convey accurate information but also align seamlessly with the perceptual and cognitive processes of each user, thereby fostering a more inclusive and user-centric design approach.

Although our primary aim is to uncover the variety of cross-modal mapping relationships and we have successfully identified various patterns and their proportions, it is also valuable to develop a general function by comparing commonly used mathematical models. Expanding our research to include a comparative analysis of these models could provide deeper insights and strengthen the robustness of our findings. Since the range of fitting functions tested was not exhaustive, further testing is necessary to determine which function or set of functions best captures the data. This is particularly important given the variability in the data due to individual differences. Future studies should expand the participant pool and increase the number of trials each participant completes. This approach will ensure a stable function can be determined, enabling more sophisticated and accurate model comparisons.

Additionally, there are limitations concerning the hearing requirements and device control in the audio experiments conducted online. We lack details about the equipment used, such as whether participants used free-field, headphones, or earphones. Despite emphasizing the importance of hearing ability during recruitment and at the experiment’s start, some participants with hearing impairments might have participated, potentially affecting their perception of frequency and volume. Moreover, volume can influence the perceived pitch of sound, leading to an association between higher pitch and higher volume [47]. However, since volume control was emphasized at the beginning, the experimental conditions for each participant can be assumed consistent. Although individual differences are unlikely to significantly impact the overall results, these uncontrolled variations could have contributed to different mapping patterns (i.e., positive or negative relationships between frequency and distance). Future studies should conduct these experiments in a controlled lab environment to achieve a level of control comparable to that of the tactile experiments.

The findings of this study have broad applicability, particularly in establishing foundational relationships for sensory substitution devices. As previously mentioned, there are numerous sensory substitution devices designed to assist individuals with visual impairments. By incorporating the presented new principles of cross-modal matching, these devices could enable users to gain a more accurate understanding of distances and changes in distance through the natural reception of auditory pitch cues or vibrotactile cues. The insights obtained from this research can extend beyond assisting visually impaired individuals and be leveraged in various fields, such as augmented reality (AR), virtual reality (VR), and teleoperation, where, although vision takes center stage, audio and tactile sensations play a complementary role. Given that users typically possess some baseline understanding of their virtual environment, audio and tactile feedback serve as augmentation tools to enhance the overall user experience. In this context, this research on cross-modal perception offers a valuable foundation for leveraging audio frequencies and vibrotactile feedback to enhance the perception of visual distances quickly and naturally. Furthermore, sensory substitution or augmentation devices that can be calibrated to the individual would enable customization and personalization of the user experience, which can improve the accuracy and comfort of the sensory experience for users and enhance accessibility, usability, and overall user satisfaction, catering to a diverse range of user needs. Meanwhile, adjusting these devices to suit each person’s unique sensory requirements, preferences, and abilities through calibration ensures that the devices are more effective and adaptable.

Future research will be important to apply these fundamental relationships to real-world applications, thereby bridging the gap between theoretical insights and practical implications. For example, participants could be actively engaged in navigating through specially designed environments while blindfolded, simulating situations that require the integration of sensory information to make informed decisions and safely traverse obstacles. This research will not only enhance understanding of cross-modal perception but will also contribute valuable data for the development of assistive technologies and tools geared toward individuals with visual impairments. Ultimately, the goal is to inform the design of more effective and user-friendly aids that can empower individuals to navigate their environments with greater independence, confidence and safety.

Additionally, future research should explore the consistency of cross-modal matching relationships in participants who engage in both auditory-vision and vibrotactile-vision matching tasks. This investigation could provide valuable insights into the similarities and differences between these two mapping processes, thereby enhancing our understanding of how different sensory modalities interact and influence each other. Such a comparative analysis could reveal underlying mechanisms and potentially lead to improved applications in areas such as sensory substitution, rehabilitation, and the design of multimodal interfaces.

## Conclusion

In this work, the vibrotactile frequency-to-visual distance and auditory frequency-to-visual distance mapping relationships were explored by allowing participants to freely match visual distance to frequencies. The findings indicate that a negative relationship was dominant for the majority of participants and a positive relationship existed for a significant minority. These results are consistent with our previous study concerning the inverse visual distance-to-auditory frequency and visual distance-to-vibrotactile frequency relationships ( [37]). In both cases, the majority correlation pattern was negative and the positive correlation pattern was a substantial minority response. Based on these results, we can conclude that the forward and inverse processes in cross-modal matching are similar in nature and capture fundamental human perceptual mapping behaviours. However, it was noted that individual differences need to be taken into account when designing new SSDs. Additionally, these studies illustrated the potential uses of both forward and inverse cross-modal mappings in various contexts, particularly in the areas of sensory substitution and augmentation. We expect these results to aid in the creation of feedback cues and assistive devices that are more natural and eﬃcient, opening the door to better user experiences and increased eﬃcacy across a range of sensory substitution and enhancement applications.

## Supporting information

A demonstration of screenshots of various webpages, such as the participant information sheet, experiment preview, electronic consent form, matching study webpages, and debrief, is included in the supplementary material. (DOCX)
