# Supplementary material for: Auditory and tactile frequency mapping for visual distance perception: A step forward in sensory substitution and augmentation
Source: PLoS One. 2025 Mar 3;20(3):e0318354. doi: 10.1371/journal.pone.0318354 (PMC11875370; doi:10.1371/journal.pone.0318354)

Designed experiment websites mainly include participant information sheet, experiment preview, electronic consent form, matching study webpages and debrief and images of these webpages were shown below:

1. Participant information sheet


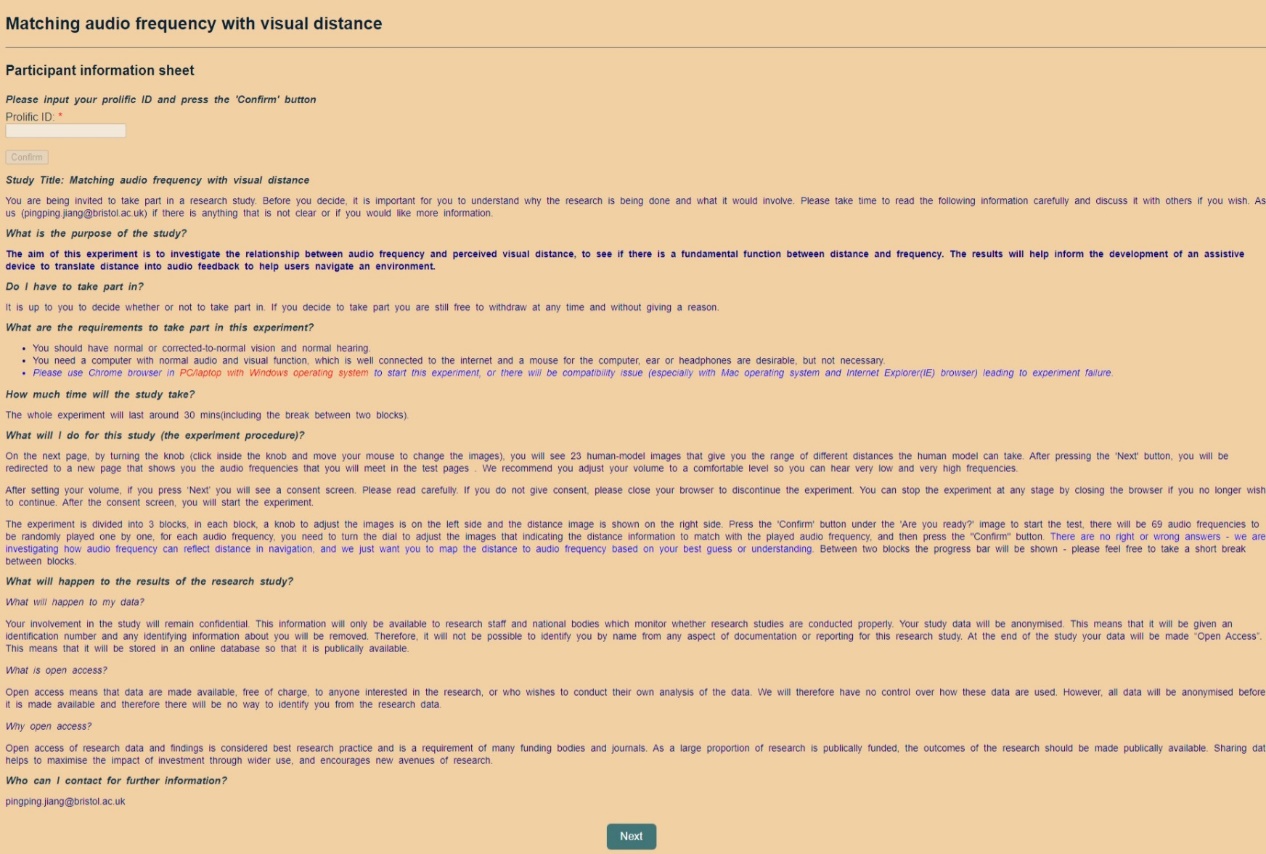


1. Image preview


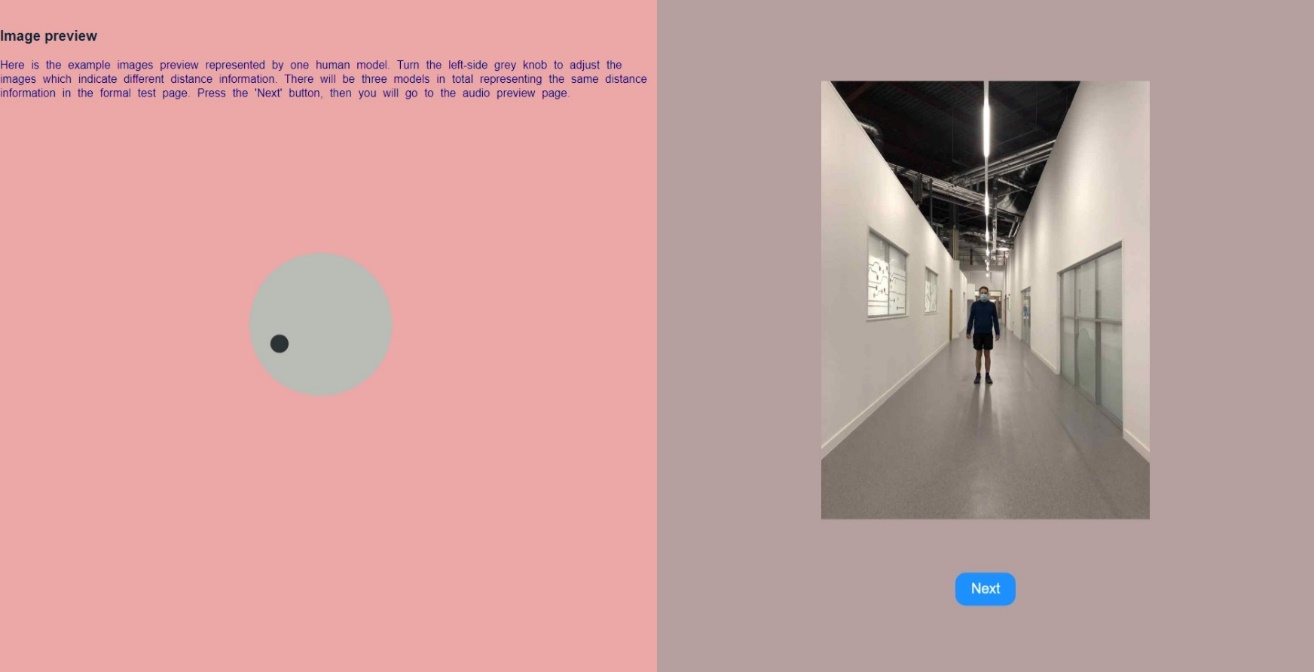


1. Audio preview


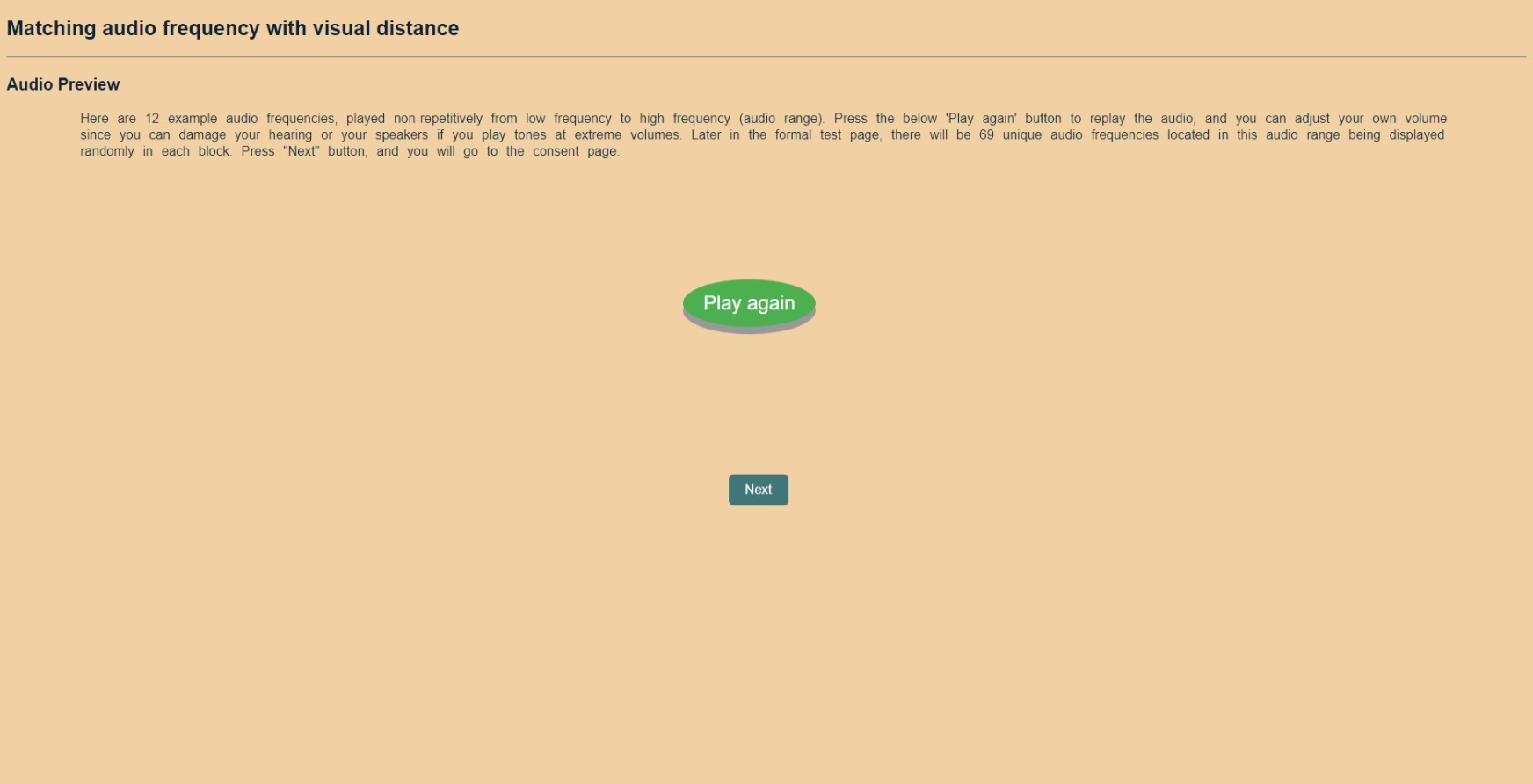


1. Consent


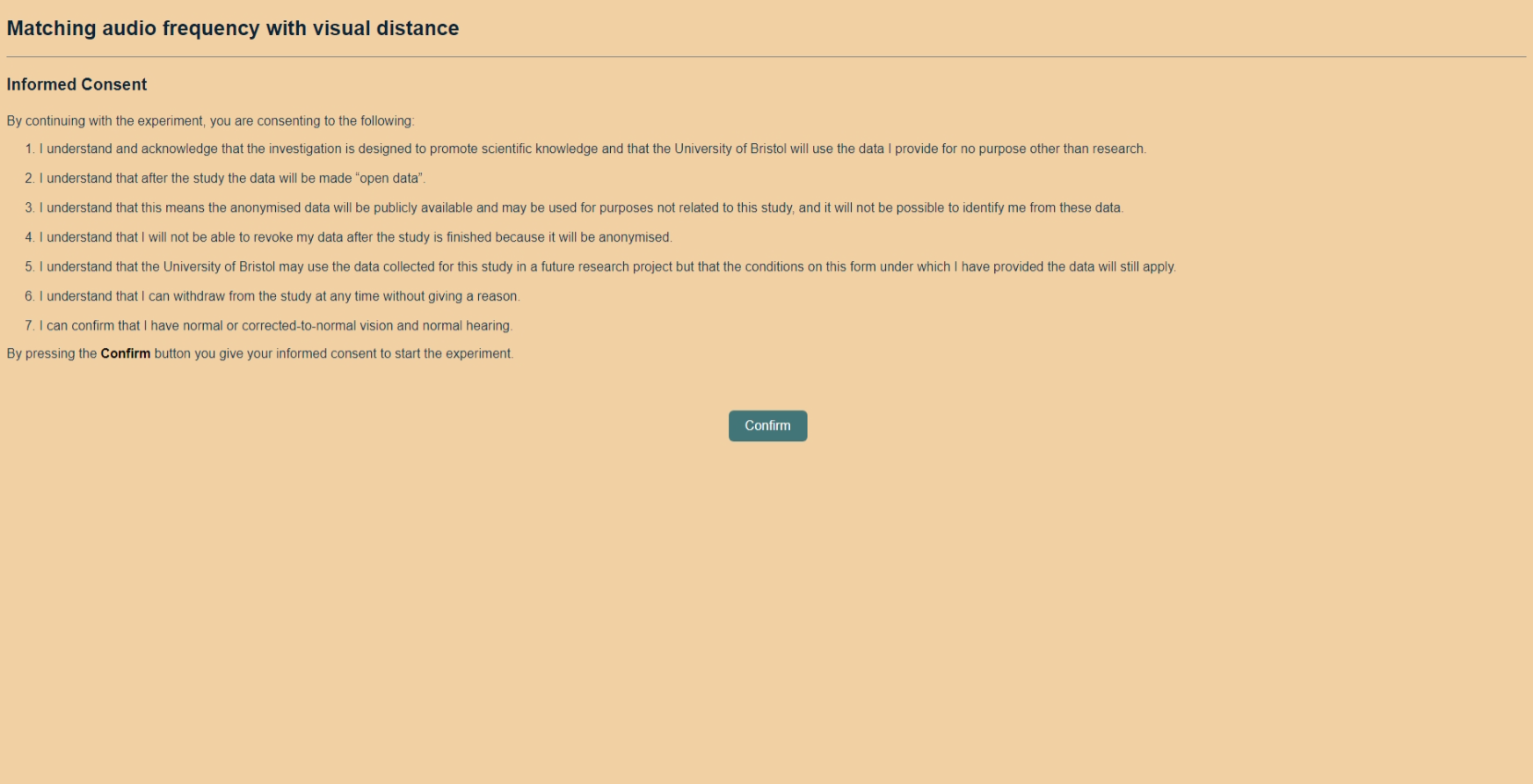


1. Matching study


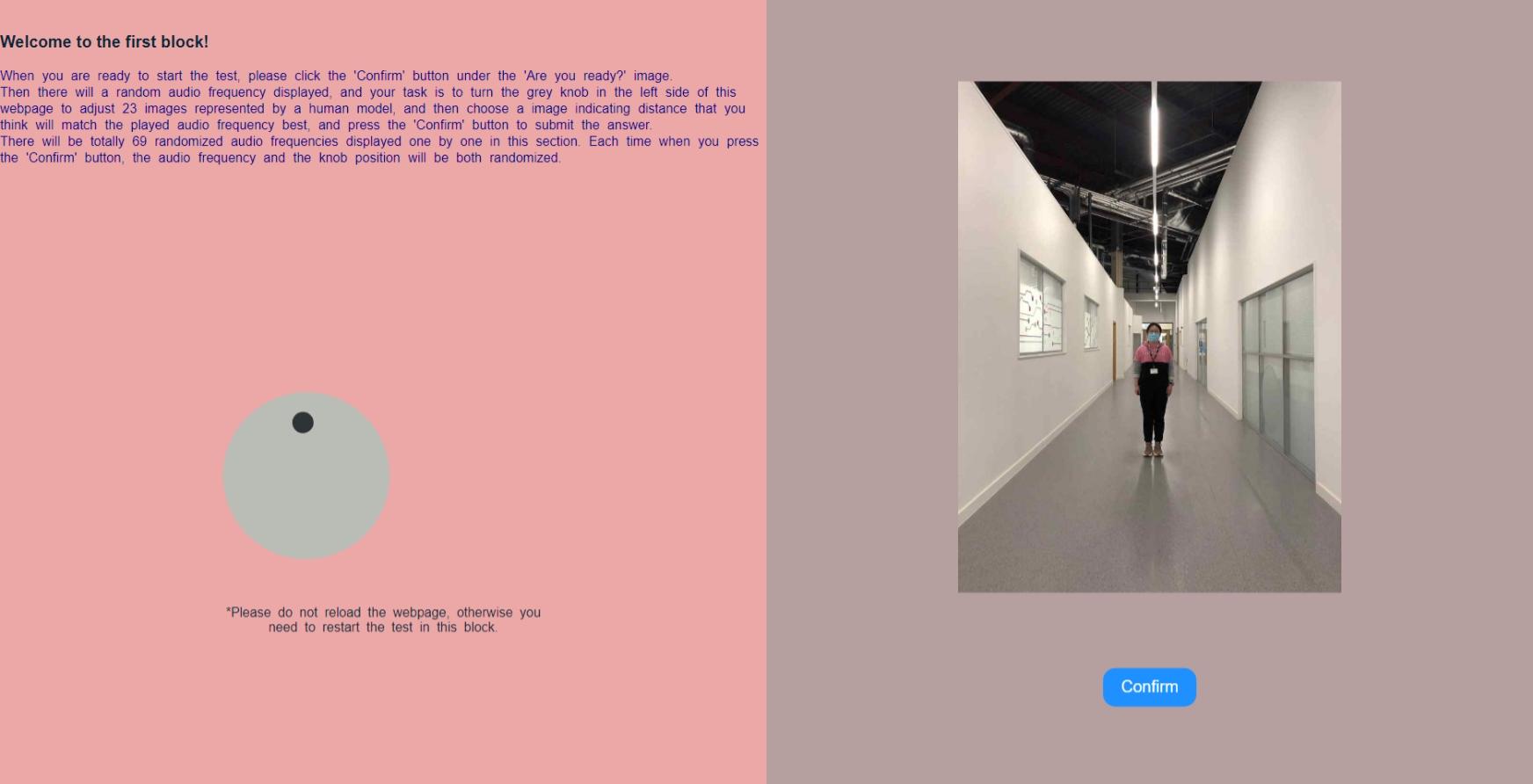


1. Debrief


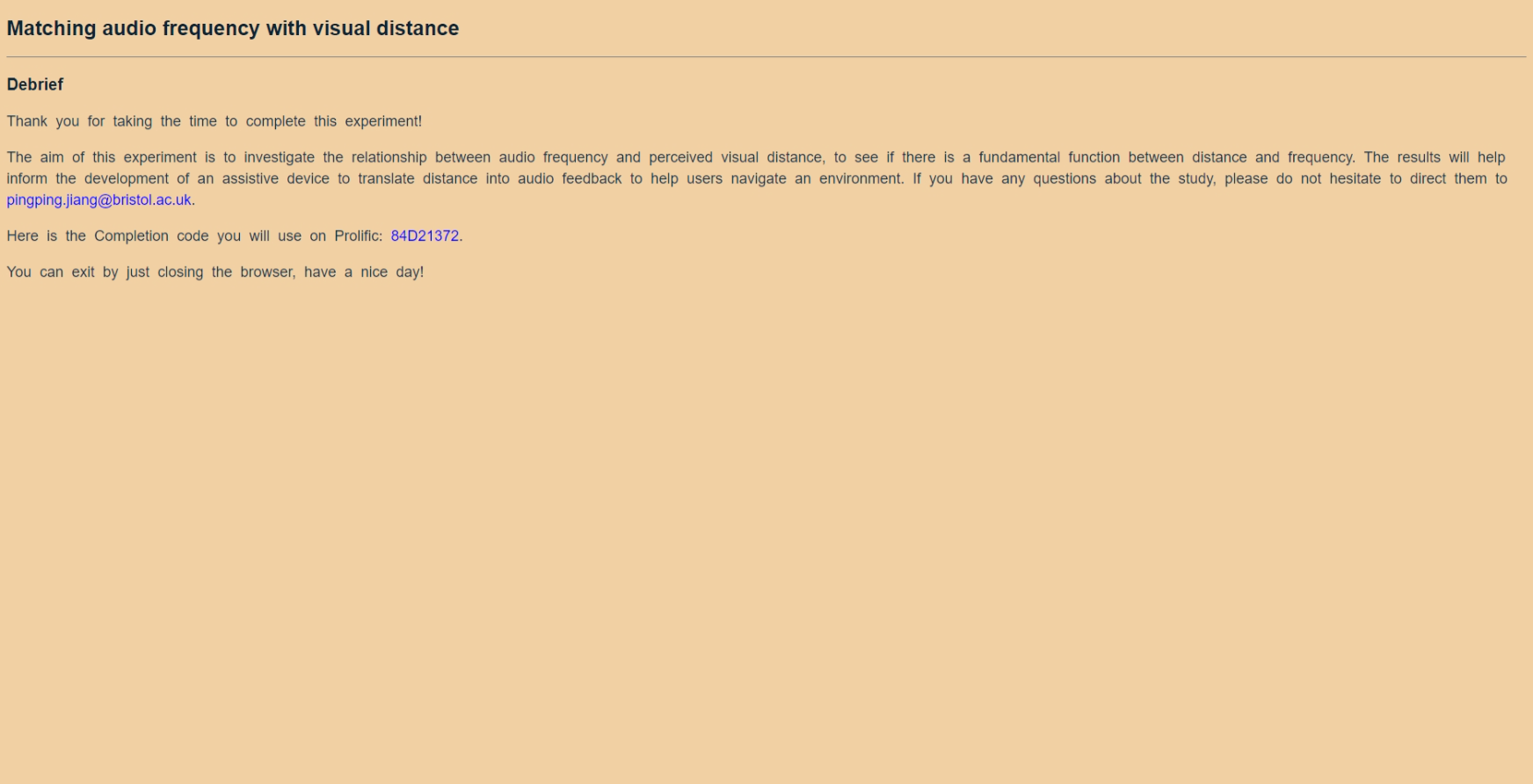

Supplement: A demonstration of screenshots of various webpages, such as the participant information sheet, experiment preview, electronic consent form, matching study webpages, and debrief, is included in the supplementary material. — (DOCX) [file pone.0318354.s001.docx]
